# Cross-Kingdom RNA Transport Based on Extracellular Vesicles Provides Innovative Tools for Plant Protection

**DOI:** 10.3390/plants13192712

**Published:** 2024-09-27

**Authors:** Yujin Zhao, Yanguang Zhou, Jingyan Xu, Sen Fan, Na Zhu, Qingling Meng, Shijie Dai, Xiaofeng Yuan

**Affiliations:** School of Life Sciences, Zhejiang Chinese Medical University, Hangzhou 310053, China; zhaoyujin326@163.com (Y.Z.); zhouyanguang07@163.com (Y.Z.); 15824357597@163.com (J.X.); fansen409@gmail.com (S.F.); zhuna85@163.com (N.Z.); 15192866713@163.com (Q.M.); dsj2513140@163.com (S.D.)

**Keywords:** extracellular vesicles, RNAi, sRNA, pathogens, plant protection

## Abstract

RNA interference (RNAi) shows great potential in plant defense against pathogens through RNA-mediated sequence-specific gene silencing. Among RNAi-based plant protection strategies, spray-induced gene silencing (SIGS) is considered a more promising approach because it utilizes the transfer of exogenous RNA between plants and microbes to silence target pathogen genes. The application of nanovesicles significantly enhances RNA stability and delivery efficiency, thereby improving the effectiveness of SIGS and further enhancing plant resistance to diseases and pathogens. This review explores the role of RNAi in plant protection, focusing on the cross-kingdom transport of small RNAs (sRNAs) via extracellular vesicles. It also explores the potential of nanotechnology to further optimize RNA-based plant protection, offering innovative tools and methods in modern plant biotechnology.

## 1. Introduction

Each year, 1 to 1.5 million pathogens are estimated to invade per hectare of soil, compounded by crops being parasitized by various fungi, nematodes, and other organisms [1]. This leads to environmental degradation and increased pest and disease incidence, resulting in significant economic losses in agricultural production [2]. To address these challenges, integrated pest management strategies are crucial for improving crop yield and quality. Currently, chemical pesticides and fungicides dominate pest control methods, but their excessive use leads to environmental pollution [3]. Consequently, scientists are increasingly focusing on the development and application of biopesticides, including the exploration of innovative technologies such as gene editing, to establish sustainable crop protection systems.

An effective strategy to control pathogens involves leveraging naturally occurring mechanisms within host–pathogen interactions. Among these, RNAi stands out as a long-established key regulatory mechanism in organisms [4]. RNAi employs a highly efficient and conserved gene regulatory mechanism that utilizes various small RNA (sRNA) populations to silence target genes in a sequence-specific manner [5]. These sRNAs can be transferred between plants and interacting organisms, thereby inducing interspecies gene silencing. Extensive research has confirmed that the RNAi pathway plays a crucial role in plant growth and development and responses to external stresses throughout various stages [6]. Specifically, in 75 related studies, RNAi through host-induced gene silencing (HIGS) technology achieved an average efficacy of 90% in virus control, while in studies on fungi, it showed an average control efficacy of 59%, particularly against parasitic and necrotrophic fungi such as *Fusarium graminearum* [6,7]. In studies on insects, the RNAi response varied among different insects, with an average control efficacy of 50% [7,8]. These studies demonstrate the significant effectiveness of RNAi technology in controlling various pathogens and pests, further supporting its broad application prospects in the field of plant protection.

In the RNAi pathway, the use of exogenous RNA, such as double-stranded RNA (dsRNA) and microRNA (miRNA), can significantly boost plant resistance to pathogens by inducing post-transcriptional gene silencing (PTGS). Application of exogenous dsRNA has been shown to decrease the virulence of pathogens on untreated distal leaves, enabling systemic spread within the plant [9], and affecting gene expression in neighboring plants [10]. However, the effectiveness of sRNAs, whether exogenous or endogenous, is impacted by their stability and delivery efficiency.

Despite the widespread phenomenon of cross-species RNA transport, the specific mechanisms by which RNA moves through the extracellular spaces of different organisms remain unclear. Extracellular vesicles (EVs) act as carriers of RNA, facilitating the transfer of sRNAs between different species, and effectively protecting and delivering these molecules while extending their stability [11,12]. EVs play a crucial role in interacting with plants and pathogens by transporting RNA to target organisms, triggering gene silencing, and inhibiting pathogen infection [12]. EVs enhance RNA delivery efficiency, allowing RNA to spread within and between plants, enabling cross-individual gene regulation and systemic protection. EV-RNAi (Extracellular Vesicle-mediated RNAi) is thus emerging as a promising tool for plant protection. The cross-species transfer of small RNAs via vesicles has been demonstrated, and artificial nanovesicles further improve RNA stability and delivery. These customizable nanovesicles can be optimized for specific pathogens or plants, paving the way for innovative RNAi applications in plant protection.

In recent years, research utilizing nanotechnology to enhance the efficiency and stability of RNAi has made significant progress, further advancing the application of EV-mediated RNAi. Consequently, EV-mediated RNAi strategies provide a safe, environmentally friendly, and sustainable method for plant protection. This paper discusses key factors for effective RNAi-based plant protection strategies, the mechanisms, and applications of RNAi in plant–pathogen interactions, and emphasizes the necessity of nanovesicles in RNA transport. By deepening the understanding of RNAi mechanisms and their practical agricultural applications, this paper aims to provide recommendations for future research to strengthen plant protection.

## 2. Regulation of Plant Disease Resistance through RNAi

RNAi plays a crucial role in regulating endogenous gene expression and defending against foreign nucleic acids, which are evolutionarily conserved processes [13,14]. Studies have shown that the plant RNAi pathway significantly influences plant immune responses by regulating the expression of transcription factors and other endogenous genes. For example, in rice, miR169 targets the transcription factor NF-YA to suppress resistance to rice blast [15], while the invasion of *Magnaporthe oryzae* suppresses the expression of miR164a, thereby relieving its repression of the transcription factor OsNAC60, activating a downstream defense gene expression, and enhancing rice resistance [16]. Other small RNAs (such as dsRNA and siRNA) also play important roles in regulating the expression of endogenous genes in plants. In *Arabidopsis thaliana*, dsRNA silences genes that negatively regulate anthocyanin accumulation, leading to increased anthocyanin content [17], while the 24-nucleotide P4-siRNA regulates the expression of endogenous genes by inducing DNA methylation in specific regions of the genome [18]. In terms of defending against exogenous nucleic acids, RNAi effectively strengthens plant immune defenses by silencing key pathogenic genes. Meng et al. found that in tomatoes, miR1001 targets key genes of *Botrytis cinerea*, inhibiting its spore germination and pathogenicity [19]. Thus, it is evident that RNAi plays multiple defensive roles in plant immunity.

**Figure 1:**
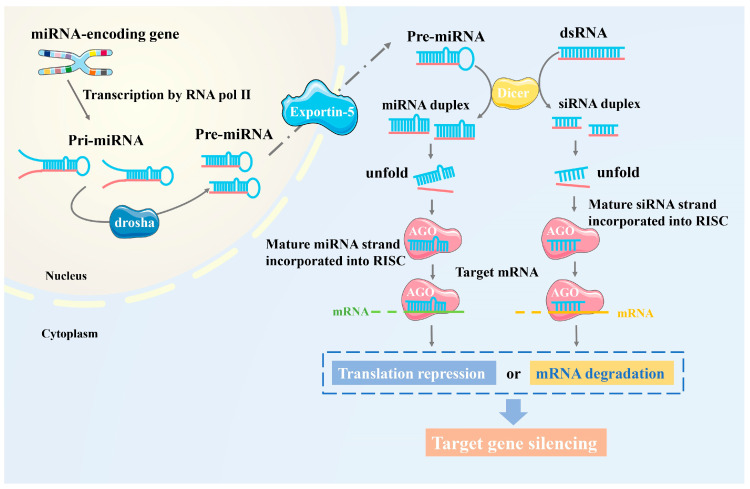
Illustration of the RNAi pathway overview. Different types of small RNA precursors are processed by Dicer proteins into siRNA or miRNA. One strand is loaded into Argonaute proteins, forming the RISC complex, which recognizes and binds to target genes, activating regulatory mechanisms such as mRNA degradation or translation repression to inhibit protein synthesis, ultimately leading to gene silencing. Drosha, a ribonuclease enzyme that processes pri-miRNA into pre-miRNA in the nucleus; Exportin-5 transports pre-miRNA from the nucleus to the cytoplasm for further processing; AGO stands for Argonaute protein, a protein family that binds to small RNAs and guides them to target mRNAs for gene silencing. Red represents the original RNA strand, blue the processed RNA (miRNA or siRNA). Green dashed mRNA is untranslated, while orange dashed mRNA is degraded or translation-repressed.

In most eukaryotes, the RNAi pathway silences target genes in a sequence-specific manner by producing distinct populations of sRNAs [20]. These sRNAs bind to Argonaute (AGO) proteins in a duplex form; the passenger strand is discarded, and the guide strand remains in the AGO protein, forming a mature RNA-induced silencing complex (RISC). The mature RISC recognizes and binds to complementary sequences of target genes through the guide strand [21]. Based on their origin and biogenesis, sRNAs are classified into siRNAs and miRNAs. siRNAs mainly derive from transposable elements, transgenes, or exogenous transcriptional genes, originating from dsRNA, and are fully complementary to their target mRNAs [22]. miRNAs are endogenous transcriptional genes purposefully expressed within the cell and are processed from pre-miRNAs with a hairpin structure. Pre-miRNAs are cleaved to produce duplex miRNAs, with one mature single-stranded miRNA retained in the RISC complex, binding to complementary mRNA sites to regulate gene expression through base pairing [23]. Although their biogenesis mechanisms differ, both siRNAs and miRNAs are processed by Dicer, bind to AGO proteins, and trigger target gene silencing to perform biological defense functions (Figure 1). siRNAs and miRNAs can cause translation repression, where the production of proteins from mRNA is blocked, or mRNA degradation, where the mRNA is broken down, preventing its translation. In the case of translation repression, siRNAs and miRNAs bind to complementary sequences in the target mRNA, often in the 3′ untranslated region (3′ UTR), thereby blocking ribosome assembly or progression along the mRNA, preventing translation into proteins [24,25]. In addition to post-transcriptional gene silencing, siRNA can also participate in transcriptional gene silencing through RNA-directed DNA methylation (RdDM). In this process, siRNA guides AGO proteins (such as AGO4) to specific DNA regions, recruiting DNA methyltransferases (such as DRM2) to add methyl groups to promoter regions, thereby inhibiting transcription.

The importance of sRNA-mediated RNAi mechanisms in eukaryotes has been widely validated, and there have been numerous studies on their role in plant–pathogen interactions [26,27]. Many plant endogenous sRNAs are involved in the regulation of plant–pathogen interactions and immune responses. These sRNAs can be transmitted between plants and pathogens, silencing genes in the interacting organisms [2,28]. In a study on Verticillium wilt, a soil-borne fungal disease of cotton (*Gossypium hirsutum*), it was found that cotton can transfer its conserved miRNAs, miR159 and miR166, into the pathogenic fungus *Verticillium dahliae* cells, thereby inhibiting the virulence of *V. dahliae* [29]. Additionally, two tasiRNAs derived from Arabidopsis TAS1 and TAS2 can target *B. cinerea* genes. Plants with high expression of these tasiRNAs show reduced susceptibility to *B. cinerea*, whereas *B. cinerea* strains with mutations in the tasiRNA target genes exhibit decreased pathogenicity [30].

RNAi is a bidirectional process where cross-kingdom sRNAs can be transmitted between plants and pathogens, regulating gene expression in the recipient cells [20,31] (Table 1). In the model plant *A. thaliana* and the fungal pathogen *B. cinerea* system, fungal sRNAs enter host cells and silence plant immunity genes, weakening host immunity to promote fungal infection [3,32]. In response, Arabidopsis secretes exosome-like extracellular vesicles that transport plant sRNAs into the fungus, targeting genes essential for fungal virulence [32]. Similarly, other fungi, oomycetes, and other plant pathogens such as *Fusarium oxysporum* [33,34], the oomycete *Hyaloperonospora arabidopsidis* [35], and the rust fungus *Puccinia striiformis* [36,37] all send sRNAs into plant host cells to target and silence the immunity genes of plants. Conversely, plant hosts such as cotton [29], tomato [19,38], and wheat [39] also produce sRNAs that can silence pathogen virulence-related genes, thereby inhibiting infection (Table 1).

Notably, although bacteria lack traditional RNAi mechanisms, they can regulate gene expression at multiple levels through other RNA regulatory pathways such as antisense RNA, functionally resembling eukaryotic RNAi [43,44].

In plant–virus interactions, RNA viral genomes form partially complementary hairpin structures, and dsRNA intermediates produced during viral replication, as well as dsRNA formed by the bidirectional transcription of DNA viruses, can be recognized by the host plant’s DCL4 protein, generating virus-derived small interfering RNAs (vsiRNAs). The vsiRNAs, incorporated into AGO protein complexes, base pair with viral RNA, leading to target cleavage or translational repression [45,46]. In addition to directly targeting viral genes, plants can also regulate endogenous gene expression to participate in antiviral defense responses. For example, when rice (*Oryza sativa*) is infected by a virus, the AGO18 protein can bind miR168 and miR528, thereby modulating rice’s antiviral capability [47]. Similarly, in rapeseed (*Brassica rapa*), miR1885 targets the resistance genes BraTNL1 and BraTIR1, regulating the sensitivity of rapeseed to turnip mosaic virus (TuMV) and participating in plant immunity [48]. During the arms race between host and virus, many viruses have evolved various viral suppressors of RNA silencing (VSR) to counteract the host plant’s antiviral RNAi [49,50]. The discovery of VSR proteins encoded by viruses further underscores the importance of RNAi in the host’s antiviral process.

RNAi, as a naturally occurring regulatory and defense mechanism, can also effectively protect crops by specifically targeting and silencing key genes related to pest growth, development, or reproduction [51]. For example, research by Zhang et al. demonstrated that silencing miR-7-5P can reduce *Nilaparvata lugens* damage to rice by 40–50% and decrease its reproduction by 41% [52]. Additionally, expressing ACT dsRNA in plants targeting the *Leptinotarsa decemlineata* (a notorious agricultural pest) resulted in 100% insect mortality [53]. These data clearly illustrate the significant impact and potential of RNAi in plant pest control. However, despite the widespread occurrence of cross-species RNA transport, the precise mechanisms by which RNA moves through the extracellular spaces of different organisms remain unclear.

## 3. Exogenous RNA and Environmental RNAi

Exogenous RNA, such as synthetic siRNA or dsRNA, can be introduced into organisms through methods like injection or soaking [54]. These RNAs mimic endogenous sRNAs and utilize the RNAi mechanism to induce gene regulation between plants and their interacting organisms [55]. Evidence provided by Federico Betti and colleagues supports the suppression of target genes miR156 and miR399 through exogenous miRNA signals, which may be transferred from the roots to the aerial parts of the plant via the xylem after absorption [10]. On the leaf surface, exogenous RNA can be directly absorbed by fungal cells or first taken up by plant cells and then transferred to fungal cells [46,56]. Additionally, local application of dsRNA on leaves has been shown to reduce the virulence of pathogens on untreated distal leaves, indicating that synthetic dsRNAs can systemically spread throughout the plant [9]. This suggests that exogenous RNA plays a significant role in plant-to-plant communication and may have great potential in environmental RNAi applications.

Unlike the exchange of sRNAs between plants and pathogens, exogenous RNA can trigger post-transcriptional gene silencing (PTGS) in recipient plants through environmental mediation, and influence gene expression in neighboring plants via inter-plant transfer [10,57]. For example, spraying dsRNA which targets specific pathogen genes on plants can induce an RNAi response in the recipient plants [55]. Can RNAi be transmitted between neighboring plants or among different species within a plant community? Currently, there is evidence of miRNA communication between adjacent plants, with experiments showing that plants can absorb miRNAs produced by neighboring plants, leading to miRNA transfer between plants and inducing PTGS in the receiving plants [9]. This indicates that miRNA mobility is not limited to the translocation of RNA signals within plants but also occurs between plants and pathogens/parasitic plants, suggesting that miRNAs may play a significant role in communication between different organisms [58]. However, these interactions require close contact between the cells of the miRNA-producing and miRNA-receiving plants. Although the mechanisms by which miRNAs are secreted from plants into the external environment and exert their effects require further research, it is acknowledged that environmental RNAi can spread throughout a plant community, forming a collective defense response. This is of great significance for highly efficient and sustainable crop protection.

In conclusion, RNA interference (RNAi) plays a crucial role in plant disease resistance, with both endogenous and exogenous mechanisms offering extensive research and application prospects. Moving forward, we will explore the pathways of cross-kingdom RNA transport and the role of extracellular vesicles in this process.

## 4. Vesicle-Mediated RNAi-Based Cross-Kingdom Trafficking

### 4.1. Intercellular Communication Based on Extracellular Vesicles

EVs, as an important tool for intercellular communication, have received extensive attention in various biological research fields in recent years. These vesicles play key roles in transporting waste, toxins, and nutrients, precisely delivering immune effectors, and serving as carriers for RNA silencing [59,60]. In animal systems, various types of mammalian cells such as adipocytes [61], cancer cells [62,63], and immune cells [64,65] can transmit miRNAs and other functional RNA molecules through EVs, thereby regulating gene expression and physiological activities in target cells. In plant systems, these vesicles not only participate in internal cellular communication but can also cross kingdoms to be transferred to pathogen cells. For instance, *A. thaliana* transfers sRNAs via EVs to the fungal pathogen *B. cinerea*, silencing its virulence genes and reducing the pathogen’s ability to cause disease [66]. Additionally, some bacteria, algae, and nematodes can use EVs to transfer RNA to interacting microorganisms or host tissues [67,68,69]. These studies highlight that EVs are major facilitators of sRNA transport between various animal, plant, or microbial cells, even though the exact mechanisms of sRNA transfer are yet to be fully understood [66,70].

### 4.2. RNA Components in Extracellular Vesicles

EVs can transport various types of RNA between cells, significantly impacting intercellular communication. These vesicles contain multiple RNA components, including sRNAs, mRNAs, and other non-coding RNAs [71,72]. Small RNAs, such as miRNA and small siRNA, regulate gene expression through the RNAi mechanism, participating in gene silencing and post-transcriptional regulation [73]. Messenger RNAs (mRNAs) carry genetic coding information that can be translated into proteins in recipient cells, thereby conveying gene expression instructions [74]. Additionally, EVs can contain other non-coding RNAs, such as circular RNAs (circRNAs) and long non-coding RNAs (lncRNAs), which influence gene expression and cellular functions through various mechanisms [59,75].

### 4.3. Extracellular Vesicle-Mediated RNA Transport

Extracellular vesicles (EVs) play a key role as RNA carriers in the cross-kingdom transport of small RNAs (sRNAs) between plants and pathogens, facilitating targeted gene silencing in interacting organisms. Recent studies suggest that the selective loading of sRNAs into EVs is mediated by specific RNA-binding proteins and other signaling molecules, which ensure that only specific sRNAs are encapsulated for transport to target organisms [76]. For instance, Cai et al. demonstrated that Arabidopsis plants selectively package miR168 into EVs, which are then transported to *B. cinerea* to silence its virulence genes [30]. This selectivity is critical for efficient and precise cross-kingdom RNA interference (RNAi).

In addition to enhancing RNA stability, EVs protect these molecules from degradation by extracellular RNases and improve the efficiency of RNA delivery to target cells. EVs can recognize specific target cells via surface receptors and fuse with them, further increasing the precision of sRNA transport. For example, cotton plants release miR159 and miR166 into the fungal pathogen *V. dahliae*, where they specifically target and silence fungal virulence genes [29]. This specificity in RNA delivery is crucial for reducing off-target effects and ensuring that RNAi remains an effective tool for plant protection.

Furthermore, the composition of miRNAs and other vesicle components can be customized based on specific plant traits and genetic factors [77]. For instance, in Arabidopsis, EVs have been identified to contain miRNAs and sRNAs (18–24 nucleotides) as well as tyRNAs (10–17 nucleotides) [71], indicating a complex eukaryotic RNA transport mechanism that directs RNA to specific destinations. The presence of various RNAs in exosomes suggests a sophisticated RNA transport system. However, the intricacies of how these unconventional non-coding RNAs are processed within vesicles, how they are taken up by target cells, and their impact on cellular communication remain largely unexplored.

Research on extracellular RNA transport protection based on EVs is crucial for improving RNA delivery efficiency and stability, which can then enhance the efficacy of RNAi. Moreover, plant EVs can encapsulate not only endogenous substances but also exogenous therapeutic molecules [78,79]. These vesicles are naturally rich in bioactive lipids, proteins, RNA, and other pharmacologically active molecules [80]. Their unique morphology and composition suggest the further development and application of exosome-like nanovesicles. This demonstrates the potential of EVs in plant protection.

The use of natural EVs enhances both RNA stability and delivery efficiency. Extending this technology to artificial nanovesicles further enhances the precision and durability of RNAi. Artificial nanovesicles can be engineered to mimic natural EVs and can be customized to target specific pathogens or plants, offering a more controllable and efficient means of RNA delivery. Studies have shown that using artificial vesicles to encapsulate dsRNA can significantly extend plant defenses against pathogens such as *B. cinerea* [81,82].

By optimizing EV-based RNA carriers, both natural and artificial, the precision and effectiveness of RNAi can be significantly improved. The development of artificial nanovesicles as carriers not only enhances RNA stability but also offers greater flexibility in designing plant protection strategies.

## 5. Strategies for Plant Protection Based on EV-RNAi

RNAi-based plant protection strategies are an innovative and eco-friendly approach that holds promise for reducing dependence on harmful pesticides while enhancing crop resistance to pathogens. Current research on the roles of sRNAs and EVs in plant defense against pathogens has led to the introduction of innovative RNAi-based methods [58,83].

### 5.1. Host-Induced Gene Silencing—HIGS

HIGS is a strategy that involves expressing homologous dsRNA or siRNA in host plants to silence genes in pests or pathogens. In HIGS, sequences from pest or pathogen genes are inserted into the host plant’s genome, allowing the expression of pathogen-specific dsRNA or sRNA, which then targets and degrades homologous transcripts [84,85]. HIGS has been proven effective against various agricultural threats, including nematodes, fungi, viruses, and viroids, demonstrating its broad applicability [14,86,87]. Additionally, it has been successfully used to protect major crops such as wheat, barley, and soybeans from various plant pathogens, including *F. oxysporum*, *B. cinerea*, and *Alternaria alternata* [10,14].

These examples indicate that HIGS is an effective strategy for reducing reliance on chemical pesticides.

The success of HIGS is closely related to the quality and quantity of small RNAs (sRNAs) transported from plant cells [88] and the absorption of these sRNAs by pathogens or pests [7]. The stability and efficient delivery of dsRNA and siRNA within plant cells are critical factors. Off-target effects may also impact the normal physiological functions of plants and pose ecological safety concerns. Additionally, the use of transgenic plants may be subject to strict regulations and public opposition in some regions, limiting the widespread application of HIGS. Despite these issues, the role of HIGS in plant protection is undeniable. However, there is a need for a more efficient, safer, and more convenient plant disease management strategy.

### 5.2. Spray-Induced Gene Silencing—SIGS

SIGS has emerged as a promising alternative to HIGS in recent years. SIGS involves the external application of dsRNA [9]. This approach helps to develop an RNAi-based, environmentally friendly plant protection strategy that does not involve genetically modified organisms. In SIGS, pathogen-targeting RNA is directly sprayed onto plants, providing a direct method for defending crops against diseases. For example, spraying dsRNA targeting the Zucchini yellow mosaic virus (ZYMV) genome can protect plants from viral infection [89]. SIGS can reduce the biomass accumulation of the soybean rust fungal pathogen by up to 75% [90].

Additionally, the application of dsRNA not only affects the directly treated areas but also impacts distant, untreated parts of the plant. This indicates that dsRNA sprayed on the surface can be absorbed into plant tissues and transported internally, allowing the silencing molecules to reach remote areas [91,92]. This mobility highlights the potential of SIGS to develop a new type of RNA-based fungicide. Unlike traditional fungicides, RNA’s rapid degradation in the soil minimizes long-term negative environmental impacts. However, the success of the SIGS method largely depends on the efficient uptake of dsRNA by the pathogen. The instability of RNA molecules significantly limits the application of this approach. To overcome this issue, researchers are exploring the use of carriers such as nanoparticles and nanovesicles to protect RNA molecules, enhancing their stability and delivery efficiency [3]. Additionally, ensuring the effective uptake of RNA molecules and their functionality in target cells is a critical challenge that needs to be addressed. Overall, while SIGS holds significant potential, its successful application depends on overcoming difficulties related to RNA stability and efficient delivery.

## 6. Nanovesicles Promote SIGS as RNA Carriers

As mentioned above, RNA molecules are highly unstable and susceptible to environmental factors such as rainfall, UV light, and enzymatic degradation, significantly reducing SIGS potency and making RNAi effects difficult to maintain over time [93]. During the dsRNA action process, extracellular nucleases secreted by the host plant or pathogen degrade the dsRNA, weakening its RNAi efficacy and shortening its persistence within the plant [94,95]. Enhancing cellular uptake (especially for plants that cannot directly absorb naked dsRNA) and improving the surface stability of dsRNA will facilitate the widespread application of RNAi technology and its transition from the lab to the field.

The use of nanovesicles significantly improves the stability and delivery efficiency of dsRNA, thereby enhancing the effectiveness of spray-induced gene silencing (SIGS) and further strengthening plant resistance to diseases and pathogens. Spraying naked dsRNA on tomatoes protected *B. cinerea* for only 7 days, indicating its rapid degradation [96]. However, Qiao et al. confirmed that dsRNA encapsulated in artificial nanovesicles substantially increases RNA stability on grape leaves, extending the duration of disease resistance to 21 days [81]. Additionally, Islam et al. used bacterial minicells to deliver dsRNA, effectively delaying its degradation and extending the protection of strawberries against gray mold infection to 12 days [97]. These results highlight the crucial role of nanovesicles in enhancing the SIGS strategy.

**Figure 2:**
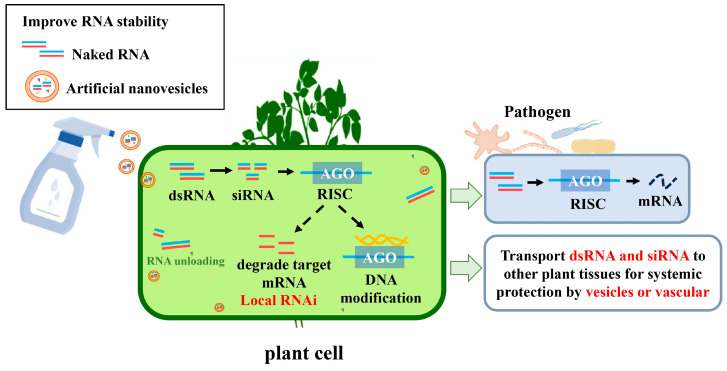
RNA nanovesicles improve SIGS crop protection efficiency. Using artificial nanovesicles to enhance RNA stability, exogenous double-stranded RNA (dsRNA) is encapsulated in vesicles, sprayed onto the plant surface, then absorbed and transported within plant cells. Dicer enzymes process dsRNA into siRNA, binding to Argonaute (AGO) proteins, forming the RNA-induced silencing complex (RISC). RISC recognizes and binds to specific target mRNAs, leading to their degradation or translation inhibition. Some siRNAs can also induce DNA modification through methylation, leading to the silencing of target genes. dsRNA or siRNA can be transported via vesicles to pathogens, silencing their virulence genes. After being processed in the cell, dsRNA and siRNA are transported to other tissues via vesicles or the plant’s vascular system, providing systemic protection for the plant. The red and blue colors in the figure represent paired RNA strands (dsRNA and siRNA).

Naturally, plants, animals, and microorganisms utilize extracellular vesicles to transport RNA, facilitating cross-kingdom RNA interference between hosts and pathogens or pests [30]. To combat RNA degradation, nanovesicles and other nanoparticles as RNA carriers have shown significant potential in plant protection (Figure 2). Nanovesicles are tiny vesicles composed of a phospholipid bilayer that can encapsulate and protect RNA molecules, enhancing their stability and reducing degradation in the environment [98]. More importantly, nanovesicles can be surface-modified with specific ligands to achieve targeted delivery to specific tissues or organs, thereby increasing the effective concentration of RNA [99,100,101].

Research indicates that sprayed dsRNA can be absorbed by plant cells and processed into siRNA through the plant’s RNAi mechanism, or it can remain intact and be transported to other tissues [102]. However, it is not yet fully understood what factors determine whether the absorbed dsRNA is processed into siRNA or transported to other parts of the plant. Potential factors influencing these processes include the structure and length of the dsRNA, the physiological state of the plant, tissue specificity, cell type, and enzymatic activity within the plant cells. Additionally, external environmental conditions such as temperature, light, and moisture may also affect how dsRNA is processed [103,104]. For certain pathogens and pests, if plant cells fail to correctly recognize or process exogenous dsRNA, these RNA molecules may be rapidly degraded or misdirected to non-target pathways, resulting in the inability to effectively silence key genes in pathogens or pests [55]. This significantly reduces the efficacy of SIGS. This highlights the importance of optimizing dsRNA design and enhancing its stability to ensure effective delivery and function within plant cells.

Stabilizing dsRNA using nanovesicles can effectively reduce incorrect recognition, degradation, and poor absorption of dsRNA. In some cases, when plants are infected by pathogens, nanovesicles can directly release the encapsulated dsRNA, enabling targeted delivery to the pathogen [105,106]. Additionally, the slow release of siRNA from the surface of nanoparticles in biological fluids can further enhance RNA delivery efficiency [107]. This approach not only protects the stability of dsRNA but also increases the local concentration and retention time of dsRNA within plant cells, thereby improving the efficacy of SIGS. Overall, the application of nanovesicles in stabilizing and delivering dsRNA provides new opportunities and technological means to enhance the effectiveness of RNAi-based plant protection strategies.

## 7. Discussion and Future Directions

RNAi technology demonstrates significant potential in plant disease management, especially in cross-species gene silencing using sRNAs and EVs [46,108]. HIGS and SIGS can induce gene silencing through different mechanisms. HIGS involves the transgenic expression of pathogen-specific dsRNA, which can enhance plant resistance but has certain limitations due to its transgenic nature [109,110]. As an alternative, SIGS provides a method for effective plant protection by directly spraying dsRNA onto the plant surface to silence target pathogen genes [111]. However, its success largely depends on the efficient absorption and internal transportation of dsRNA within the plant system [112]. The application of nanoparticles can significantly enhance the stability and delivery efficiency of dsRNA, thereby improving the effectiveness of SIGS. By applying multiple dsRNA molecules, combining dsRNA and siRNA molecules, or integrating them with fungicides or insecticides, plant defenses against various pathogens can be strengthened. This multifaceted approach not only enhances protective effects but also reduces the risk of pathogens developing resistance or tolerance [9,113]. Of course, efficiently loading RNA is a critical aspect, and innovative RNA loading technologies need further exploration.

From a cost-effectiveness perspective, although the initial investment in research and development and production of RNAi products—such as the synthesis and delivery systems for double-stranded RNA—is high, the long-term benefits are significant. These benefits include the reduced use of pesticides, decreased reliance on antibiotics, increased crop yields, and improved crop quality [114], thereby achieving sustainable economic and environmental benefits.

In terms of risks, RNAi technology is highly specific and exhibits low toxicity, thus posing minimal direct risks to human health. The environmental risks are primarily concentrated on non-target effects and the degradation of nanocarriers. The widespread use of RNAi technology could have long-term impacts on ecosystems, including potential off-target risks and effects on non-target species, all of which require further in-depth research. Additionally, the biodegradability of nanocarriers used in RNAi also needs to be rigorously assessed. While RNA typically degrades quickly in natural environments, synthetic nanoparticles used as carriers might not decompose as rapidly and could pose threats to non-target organisms. Therefore, comprehensive environmental and health risk assessments are crucial for ensuring the safety and sustainability of RNAi-based crop protection technologies.

In summary, although RNAi technology based on nanovesicles has shown potential for low-risk and highly sustainable management in agricultural pest and disease control, we must clarify that these conclusions are based on current scientific understanding and limited experimental studies. A thorough assessment of the long-term impacts of RNAi technology on non-target species and the entire ecosystem is still required. Overall, RNAi technology based on nanovesicles, as an alternative to traditional chemical pesticides, offers an environmentally friendly and efficient method of plant disease management. Looking forward, as more research is conducted, we believe that RNAi will play a more significant role in agricultural disease management, laying a solid foundation for achieving more environmentally friendly and efficient plant protection strategies.

## Figures and Tables

**Table 1:** RNA transfer in plant–pathogen communication.

Host Plant	Pathogen	Goal/Target of RNAi	RNA Type	Directionality of RNA Mobility	Ref.
*Lycopersicum esculentum* (tomato); *Arabidopsis thaliana*	*Botrytis cinerea*	Plants	siRNA	Pathogen–host	[32]
*Gossypoim hirsutism* (cotton)	*Verticillium dahliae*	Fungi	miRNA	Host–pathogen	[29]
*Lycopersicum esculentum* (tomato); *Arabidopsis thaliana*	*Botrytis cinerea*	Fungi	siRNA	Host–pathogen	[40]
*Arabidopsis thaliana*	*Cuscuta campestris*	Plants	miRNA	Pathogen–host	[41]
*Arabidopsis thaliana*	*Botrytis cinerea*	Fungi	siRNA, miRNA	Host–pathogen	[30]
*Arabidopsis thaliana*	*Hyaloperonospora arabidopsidis*	Plants	sRNA	Pathogen–host	[35]
*Lycopersicum esculentum* (tomato)	*Botrytis cinerea*	Fungi	miRNA	Host–pathogen	[27]
*Lycopersicum esculentum* (tomato)	*Fusarium oxysporum*	Plants	mRNA	Pathogen–host	[36]
*Arabidopsis thaliana*	*Verticillium dahliae*	Plants	miRNA	Pathogen–host	[42]
*Lycopersicum esculentum* (tomato)	*Botrytis cinerea*	Fungi	siRNA	Host–pathogen	[38]

## Data Availability

Not applicable.
